# Safety and efficacy analysis of neoadjuvant radiotherapy combined with concurrent paclitaxel plus nedaplatin versus other platinum-based chemotherapy for thoracic segmental esophageal squamous cell carcinoma

**DOI:** 10.3389/fonc.2025.1582481

**Published:** 2025-05-13

**Authors:** Qingyang Zhuang, Hui Li, Lirui Tang, Hongying Zheng, Jiancheng Li, Junxin Wu, Jinluan Li

**Affiliations:** ^1^ Department of Radiation Oncology, Clinical Oncology School of Fujian Medical University, Fujian Cancer Hospital, Fuzhou, China; ^2^ Department of Oncology, Fuzhou Pulmonary Hospital of Fujian, Fuzhou, China

**Keywords:** nedaplatin, platinum, neoadjuvant chemoradiotherapy, esophageal squamous cell carcinoma, safety analysis, efficacy analysis

## Abstract

**Background:**

Esophageal cancer is among the leading causes of cancer-related mortality in males. This study aimed to evaluate the efficacy and safety of nedaplatin (NDP) in comparison to other platinum-based (OPB) agents combined with paclitaxel and concurrent neoadjuvant radiotherapy for locally advanced thoracic segmental esophageal squamous cell carcinoma (ESCC).

**Methods:**

This single-center, retrospective cohort study was conducted in China. The primary endpoints of this study were safety and efficacy assessments. Unpaired t-tests, chi-squared tests, and Fisher’s exact tests were used to compare intergroup differences, as appropriate. Multivariate logistic regression models were used to explore the associations between postoperative outcomes and the two treatment groups. Kaplan–Meier survival curves and Cox proportional hazards regression models based on OS and PFS were used to compare the efficacy between the two groups.

**Results:**

A total of 212 patients were enrolled in this retrospective cohort study, including 79 who received NDP and 133 who received OPB (82 were treated with cisplatin, 20 with carboplatin, 19 with lobaplatin, and 12 with oxaliplatin) agents. The incidences of grade 3–4 acute radiotherapy-associated esophagitis, pneumonitis, and leukemia were significantly lower in the NDP group than in the OPB group (p = 0.02, p < 0.001, and p = 0.002, respectively). All grades of acute gastrointestinal reactions, including nausea, vomiting, anorexia, and diarrhea, were significantly more frequent in the OPB group than in the NPD group (p < 0.001, p = 0.032, p < 0.001, and p = 0.002, respectively). The Kaplan–Meier curves for overall survival (OS) and progression-free survival (PFS) showed similar results for both groups.

**Conclusions:**

The safety profile of nedaplatin may be superior to those of other platinum-based agents in terms of acute radiotherapy toxicity and postoperative side effects; however, there was no difference in the efficacy between the two groups regarding short-term prognostic tumor regression grades or long-term OS and PFS.

## 1 Introduction

Esophageal cancer is among the lea ding causes of cancer-related mortality in males, ranking tenth in the United States, with an estimated 12,880 male deaths by 2024 (1). The 5-year survival rate for esophageal cancer is alarmingly low at approximately 22% (1, 2). In Asia, male predominance is striking, with squamous cell carcinoma accounting for approximately 90% of cases (3). The R0 resection rate was only 50%, and the early recurrence rate was notably high (4). Neoadjuvant radiotherapy combined with concurrent chemotherapy has been postulated to enhance the pathological response rate and mitigate adverse events in locally advanced esophageal squamous cell carcinoma (ESCC); however, its long-term benefits remain controversial (5). Despite these interventions, recurrence rates following neoadjuvant therapy can reach 33–48% (6, 7). Consequently, there is an urgent need to develop more effective and safer treatment strategies for ESCC.

Nedaplatin (NDP), a second-generation platinum analog, has shown promise in clinical trials, with a response rate similar to that of cisplatin, a gold-standard platinum-based agent (8–10). However, the durability of response and overall survival (OS) rates with NDP have been reported to be lower than those achieved with cisplatin (11–13). Previous studies have compared NDP with other platinum-based (OPB) chemotherapies for the treatment of malignancies including ESCC (12, 14–17). For instance, Ohnuma et al. previously reported that patients with stage IB to IV esophageal cancer receiving NDP-based chemoradiotherapy achieved an 82.1% complete response rate and exhibited median progression-free and overall survivals of 41.2 months (18). Furthermore, the choice of a concurrent chemotherapy regimen in combination with NDP has been debated, with variable results reported across different patient populations and geographical regions (19–22).

This study aimed to conduct a comprehensive safety and efficacy analysis of neoadjuvant radiotherapy combined with concurrent paclitaxel plus NDP compared with OPB chemotherapy regimens for the treatment of thoracic segmental ESCC. By evaluating the short- and long-term outcomes, we aimed to determine the potential advantages of this treatment approach in terms of pathological response rates, OS, and toxicity profiles. Additionally, these findings may contribute to a better understanding of the underlying mechanisms that govern the response to neoadjuvant therapy and the development of novel therapeutic strategies tailored to the specific molecular characteristics of ESCC.

## 2 Materials and methods

### 2.1 Study design and population

This single-center, retrospective cohort study was conducted at Fujian Cancer Hospital in China. Patients diagnosed with locally advanced ESCC between January 2006 and December 2022, who underwent neoadjuvant chemoradiotherapy (nCRT) and surgical resection, were included. Patients were considered for inclusion if they met the following criteria: (1) aged ≥ 18 years, (2) clinically staged as II/III/IVA with no distant metastases at the time of initial diagnosis, (3) received complete neoadjuvant radiotherapy, (4) received neoadjuvant chemotherapy with paclitaxel plus a platinum-based drug (NDP or OPB derivatives such as cisplatin, carboplatin, laboplatin, or oxaliplatin), (5) underwent resection of the primary esophageal lesion after nCRT, and (6) had complete pathological data, including tumor regression grades according to the NCCN and JSED criteria. The exclusion criteria were as follows: (1) cervical and gastroesophageal junction tumors; (2) neoadjuvant chemotherapy with drugs other than paclitaxel and platinum; (3) severe preexisting organ disease before treatment; (4) presence of multiple primary malignancies; and (5) missing or incomplete medical records or follow-up information.

### 2.2 Cohort assignment

This study grouped patients according to different treatment regimens as exposures. The specific grouping criteria were as follows: patients who received nCRT concurrent with paclitaxel plus NDP, followed by surgical resection and lymph node dissection (NDP group), and those who received nCRT concurrent with paclitaxel plus OPB drugs, followed by surgical resection and lymph node dissection (OPB group). Neoadjuvant radiotherapy consisted of 41.4~50.4 Gy/23~28 fractions delivered using three-dimensional conformal radiation therapy or intensity-modulated radiation therapy in computed tomography (CT) simulation and conformal treatment planning. The preoperative chemotherapy agents included paclitaxel plus NDP or OPB derivatives (including cisplatin, carboplatin, cisplatin, and oxaliplatin).

### 2.3 Endpoints

The primary endpoints of this study were safety and efficacy assessments. Safety assessments included acute toxic reactions during neoadjuvant treatment until the time of surgery and postoperative evaluation metrics. Acute toxic reactions included radiation-related dermatitis, esophagitis, and pneumonitis, in addition to gastrointestinal reactions such as nausea, vomiting, neurogenic anorexia, and diarrhea. The hematological toxic effects included leukopenia, hemoglobin changes, thrombocytopenia, and an increase in the alanine aminotransferase-to-bilirubin ratio. Postoperative indicator, textbook outcome (TO), was assessed using a composite index comprising the lymph node yield, R0 resection status, length of postoperative hospital stay (PLOS), occurrence of postoperative complications, 30-day mortality, and readmission rates. The pathological tumor regression grades (TRGs) were reported by two experienced pathologists: NCCN-TRG and JSED-TRG. OS and progression-free survival (PFS) were used to assess the efficacy. OS was defined as the date from the start of surgery to the last follow-up or death from any cause. PFS was defined as the date from the start of surgery to the last follow-up or disease progression, which was determined radiologically or pathologically.

### 2.4 Statistical analyses

All statistical analyses were performed using R software (version 4.3.2). Baseline characteristics are presented based on variable types. Continuous variables with a normal distribution were expressed as means ± standard deviation (SD), while those with a skewed distribution were reported as medians with interquartile ranges. Categorical and ordinal variables were presented as proportions. Unpaired t-tests, chi-squared tests, and Fisher’s exact tests were used to compare intergroup differences, as appropriate. Multivariate logistic regression models adjusted for confounding factors were used to explore the associations between postoperative outcomes (TO, NCCN-TRG, and JSED-TRG) and the two treatment groups. Kaplan–Meier survival curves based on OS and PFS were used to compare the efficacy between the two groups. Univariate and multivariate Cox proportional hazards regression models, along with their visualized forest plots, were used to identify subgroups within the two groups that demonstrated OS or PFS benefits. Statistical significance was set at p < 0.05.

## 3 Results

### 3.1 Baseline characteristics

In this study, 357 patients with ESCC in the thoracic segment were included; 145 patients did not meet the inclusion criteria. Finally, 212 patients were included in the analysis: 79 in the NDP-based treatment group and 133 in the OPB treatment group. The baseline characteristics of the two groups and their intergroup differences are presented in **Table 1**. The mean age of the patients was 56.22 ± 8.16 years. The OPB group had a mean age of 55.14 ± 8.41 years, while the NDP group had a mean age of 58.05 ± 7.43 years (t = –2.55, p = 0.012). The sex distribution was similar in both groups, with females comprising 13.21% and 16.46% of the OPB and NDP groups, respectively, and males comprising 86.79% and 83.54% of the OPB and NDP groups, respectively (χ² = 1.16; p = 0.282). Furthermore, the body mass index (BMI) and albumin levels did not differ between the two groups (t = 0.47, p = 0.642; t = –1.57, p = 0.117, respectively). HBsAg status, primary site, hypertension, diabetes, family cancer history, smoking, drinking, weight loss, and tumor stage were similar in both groups.

**Table 1:** Baseline characteristics of locally advanced esophageal squamous carcinoma.

Variables	Total (n = 212)	OPB Group (n = 133)	NDP Group (n = 79)	Statistic	*P*
Age, Mean ± SD	56.22 ± 8.16	55.14 ± 8.41	58.05 ± 7.43	t=-2.55	**0.012**
BMI, Mean ± SD	21.88 ± 2.89	21.96 ± 2.88	21.76 ± 2.92	t=0.47	0.642
Albumin, Mean ± SD	38.72 ± 3.78	38.40 ± 3.78	39.25 ± 3.75	t=-1.57	0.117
Sex, n(%)				χ²=1.16	0.282
female	28 (13.21)	15 (11.28)	13 (16.46)		
male	184 (86.79)	118 (88.72)	66 (83.54)		
Primary Site, n(%)				χ²=0.17	0.920
Lower thorax	27 (12.74)	16 (12.03)	11 (13.92)		
Middle thorax	134 (63.21)	85 (63.91)	49 (62.03)		
Upper thorax	51 (24.06)	32 (24.06)	19 (24.05)		
HBsAg, n(%)				χ²=0.29	0.592
Negative	176 (83.02)	109 (81.95)	67 (84.81)		
Positive	36 (16.98)	24 (18.05)	12 (15.19)		
Hypertension, n(%)				χ²=2.88	0.089
No	171 (80.66)	112 (84.21)	59 (74.68)		
Yes	41 (19.34)	21 (15.79)	20 (25.32)		
Diabetes, n(%)				χ²=2.61	0.106
No	180 (84.91)	117 (87.97)	63 (79.75)		
Yes	32 (15.09)	16 (12.03)	16 (20.25)		
Family cancer history, n(%)				χ²=1.94	0.163
No	195 (91.98)	125 (93.98)	70 (88.61)		
Yes	17 (8.02)	8 (6.02)	9 (11.39)		
Smoking, n(%)				χ²=3.03	0.082
No	107 (50.47)	61 (45.86)	46 (58.23)		
Yes	105 (49.53)	72 (54.14)	33 (41.77)		
Drinking, n(%)				χ²=0.15	0.694
No	166 (78.30)	103 (77.44)	63 (79.75)		
Yes	46 (21.70)	30 (22.56)	16 (20.25)		
Weight Loss, n(%)				χ²=0.52	0.470
No	74 (34.91)	44 (33.08)	30 (37.97)		
Yes	138 (65.09)	89 (66.92)	49 (62.03)		
cStage, n(%)				χ²=0.35	0.839
II	40 (18.87)	25 (18.80)	15 (18.99)		
III	108 (50.94)	66 (49.62)	42 (53.16)		
IVA	64 (30.19)	42 (31.58)	22 (27.85)		

OPB, other platinum-based; including cisplatin, carboplatin, lobaplatin, and oxiliplatin; NPD, Nedaplatin; t, t-test; χ², Chi-square test; BMI, body mass index; SD, standard deviation.The bold values indicates that this section is statistically significant.

### 3.2 Comparison of different treatment regimes

In the OPB treatment group, 82 were treated with cisplatin, 20 with carboplatin, 19 with lobaplatin, and 12 with oxaliplatin. The clinical assessments, treatments, and pathological staging of the different treatment groups are shown in **Table 2**. The interval between neoadjuvant therapy and surgery (N and S) was similar across all treatment groups, with a mean of 5.24 ± 1.70 days. The mean blood loss during surgery of the NDP group (160.38 ± 121.73 ml) was slightly lower than that in the OPB group; however, there was no significant difference between all groups (F = 0.38; p = 0.825). The preoperative clinical evaluation revealed similar responses across the treatment groups (p = 0.286). The number of chemotherapy cycles and radiation approaches used varied slightly across the treatment groups. In addition, the tumor stage at the time of pathological diagnosis did not differ significantly between the treatment groups, including pT, pN, and pStage (p = 0.171, 0.658, and 0.683, respectively).

**Table 2:** Different interventions and clinical outcomes of locally advanced esophageal squamous carcinoma.

Variables	Total (n = 212)	NDP Group (n = 79)	Other Platinum-based Group					
			DDP (n = 82)	CBP (n = 20)	Lobaplatin (n = 19)	Oxaliplatin (n = 12)	Statistic	*P*
Interval between N & S, Mean ± SD	5.24 ± 1.70	5.44 ± 2.03	5.22 ± 1.54	5.10 ± 1.48	4.63 ± 1.30	5.17 ± 1.11	F=0.93	0.447
Blood loss, Mean ± SD	171.32 ± 112.97	160.38 ± 121.73	176.22 ± 105.48	170.00 ± 111.69	189.47 ± 125.36	183.33 ± 93.74	F=0.38	0.825
Preoperative clinical evaluation, n(%)							-	0.286*
CR	67 (31.60)	29 (36.71)	23 (28.05)	6 (30.00)	5 (26.32)	4 (33.33)		
PD	11 (5.19)	4 (5.06)	3 (3.66)	1 (5.00)	0 (0.00)	3 (25.00)		
PR	91 (42.92)	36 (45.57)	35 (42.68)	8 (40.00)	9 (47.37)	3 (25.00)		
SD	43 (20.28)	10 (12.66)	21 (25.61)	5 (25.00)	5 (26.32)	2 (16.67)		
Chemotherapy cycle							-	0.758
1	87 (41.04)	35 (44.30)	29 (35.37)	9 (45.00)	8 (42.11)	6 (50.00)		
2	109 (51.42)	40 (50.63)	42 (51.22)	10 (50.00)	11 (57.89)	6 (50.00)		
3-4	16 (7.55)	4 (5.06)	11 (13.42)	1 (5.00)	0 (0.00)	0 (0.00)		
Radiation approach							χ²=4.77	0.312
IMRT	156 (73.58)	58 (73.42)	58 (70.73)	18 (90.00)	12 (63.16)	10 (83.33)		
3DCRT	56 (26.42)	21 (26.58)	24 (29.27)	2 (10.00)	7 (36.84)	2 (16.67)		
pT Stage, n(%)							-	0.171*
T0	57 (26.89)	25 (31.65)	21 (25.61)	2 (10.00)	4 (21.05)	5 (41.67)		
T1a	8 (3.77)	7 (8.86)	1 (1.22)	0 (0.00)	0 (0.00)	0 (0.00)		
T1b	15 (7.08)	7 (8.86)	7 (8.54)	1 (5.00)	0 (0.00)	0 (0.00)		
T2	49 (23.11)	11 (13.92)	20 (24.39)	8 (40.00)	6 (31.58)	4 (33.33)		
T3	74 (34.91)	27 (34.18)	29 (35.37)	8 (40.00)	8 (42.11)	2 (16.67)		
T4a	7 (3.30)	2 (2.53)	3 (3.66)	0 (0.00)	1 (5.26)	1 (8.33)		
T4b	2 (0.94)	0 (0.00)	1 (1.22)	1 (5.00)	0 (0.00)	0 (0.00)		
pN Stage, n(%)							-	0.658*
N0	123 (58.02)	49 (62.03)	46 (56.10)	9 (45.00)	13 (68.42)	6 (50.00)		
N1	55 (25.94)	20 (25.32)	24 (29.27)	4 (20.00)	4 (21.05)	3 (25.00)		
N2	22 (10.38)	6 (7.59)	8 (9.76)	5 (25.00)	1 (5.26)	2 (16.67)		
N3	12 (5.66)	4 (5.06)	4 (4.88)	2 (10.00)	1 (5.26)	1 (8.33)		
pStage, n(%)							-	0.683*
I	83 (39.15)	37 (46.84)	30 (36.59)	4 (20.00)	7 (36.84)	5 (41.67)		
II	34 (16.04)	11 (13.92)	14 (17.07)	4 (20.00)	5 (26.32)	0 (0.00)		
IIIA	31 (14.62)	12 (15.19)	13 (15.85)	2 (10.00)	2 (10.53)	2 (16.67)		
IIIB	48 (22.64)	14 (17.72)	19 (23.17)	7 (35.00)	4 (21.05)	4 (33.33)		
IVA	16 (7.55)	5 (6.33)	6 (7.32)	3 (15.00)	1 (5.26)	1 (8.33)		

NPD, Nedaplatin; DDP, cisplatin; CBP, carboplatin; N & S, neoadjuvant therapy and surgery; SD, standard deviation; F, ANOVA; χ², Chi-square test; -: Fisher exact; *, Simulated p-value; CR, complete response; PR, partial response; SD, stable disease; PD, progressive disease; IMRT, intensity modulated radiation therapy; 3DCRT, three-dimensional conformal radiation therapy.

### 3.3 Acute toxic effects reported during neoadjuvant therapy or before surgery

Radiation-related toxic effects were common among all patients who received neoadjuvant therapy (**Table 3**). The most frequently reported was acute radiation-related dermatitis (ARD), which occurred in 83% of patients in the OPB group and 54% in the NDP group, respectively, although this difference was not statistically significant (p = 0.381). However, acute radiation-related esophagitis (ARE) was more commonly reported in the OPB group (n = 114, 85.71%) than in the NDP group (n = 64, 81.01%); this difference was statistically significant (p = 0.367). Furthermore, acute radiation-related pneumonitis (ARP) was significantly more common in the OPB group (n = 127, 95.49%) than in the NDP group (n = 52, 65.82%); this difference was also statistically significant (p < 0.001).

**Table 3:** Acute toxic effects reported during neoadjuvant therapy or before surgery.

Acute Toxic Effect	All grade			Grade 3-4		
	OPB Group (n = 133)	NDP Group (n = 79)	*P*	OPB Group (n = 133)	NDP Group (n = 79)	*P*
Radiation-related						
ARD, n(%)	83 (62.41)	54 (68.35)	0.381	6 (4.51)	6 (7.59)	0.527
ARE, n(%)	114 (85.71)	64 (81.01)	0.367	41 (30.83)	13 (16.46)	**0.02**
ARP, n(%)	127 (95.49)	52 (65.82)	**<.001**	62 (46.62)	16 (20.25)	**<.001**
Gastrointestinal						
Nausea, n(%)	78 (58.65)	25 (31.65)	**<.001**	9 (6.77)	1 (1.27)	0.136
Vomiting, n(%)	31 (23.31)	9 (11.39)	**0.032**	4 (3.01)	1 (1.27)	0.734
Anorexia, n(%)	113 (84.96)	40 (50.63)	**<.001**	10 (7.52)	1 (1.27)	0.096
Diarrhea, n(%)	38 (28.57)	8 (10.13)	**0.002**	4 (3.01)	1 (1.27)	0.734
Hematologic						
Leukemia, n(%)	125 (93.98)	66 (83.54)	**0.014**	71 (53.38)	25 (31.65)	**0.002**
Hemoglobin, n(%)	23 (17.29)	9 (11.39)	0.246	4 (3.01)	0 (0.00)	0.301
Thrombocytopenia, n(%)	14 (10.53)	3 (3.80)	0.081	2 (1.50)	0 (0.00)	0.53
ALT to bilirubin ratio increase, n(%)	11 (8.27)	1 (1.27)	0.068	1 (0.75)	0 (0.00)	1

OPB, other platinum-based; including cisplatin, carboplatin, lobaplatin, and oxiliplatin; NPD, Nedaplatin; ARD, acute radiation-related dermatitis; ARE, acute radiation-related esophagitis; ARP, acute radiation-related pneumonitis; ALT, Alanine Amino Transferase.The bold values indicates that this section is statistically significant.

Gastrointestinal toxicity was also common. Nausea was the most reported gastrointestinal symptom, with 78% of patients in the OPB group and 25% in the NDP group experiencing it, with a statistically significant difference between groups (p < 0.001). Vomiting was less common, with 31 patients (23.31%) in the OPB group and nine patients (11.39%) in the NDP group, with a difference that approached statistical significance (p = 0.032). Anorexia was significantly more common in the OPB group (n = 113, 84.96%) than in the NDP group (n = 40, 50.63%; p < 0.001). Furthermore, diarrhea was less common, with 38 patients (28.57%) in the OPB group and eight patients (10.13%) in the NDP group experiencing it, with a statistically significant difference (p = 0.002).

Hematological toxicity was also observed. Leukopenia, a decrease in the white blood cell count, was significantly more common in the OPB group (n = 125, 93.98%) than in the NDP group (n = 66, 83.54%; p = 0.014). The hemoglobin levels decreased in 23 patients (17.29%) in the OPB group and 9 patients (11.39%) in the NDP group, with no statistically significant difference between groups (p = 0.246). Thrombocytopenia, a decrease in the platelet count, occurred in 14 patients (10.53%) in the OPB group and three patients (3.80%) in the NDP group, with no statistically significant difference between groups (p = 0.081). Furthermore, an increase in the ALT-to-bilirubin ratio, an indicator of liver function abnormalities, was more common in the OPB group (n = 11, 8.27%) than in the NDP group (n = 1, 1.27%), although this difference was not statistically significant (p = 0.068).

### 3.4 Postoperative evaluation indicators

We first analyzed the differences in the surgical evaluation indicators and complications between the two groups (**Table 4**). The TO rate was higher in the OPB group (84.21%) compared to the NDP group (74.68%), although this difference was not statistically significant (χ² = 2.88; p = 0.089). However, the number of lymph node dissections (LNDs) with < 20 lymph nodes was significantly higher in the NDP group (38.35%) compared to the OPB group (51%; χ² = 3.78; p = 0.052). The R0 tumor margin rates were 90.98% in the OPB group and 98.73% in the NDP group (χ² = 3.92; p = 0.048). The PLOS was similar between the two groups, with 59.40% of patients in the OPB group and 46.58% in the NDP group having a PLOS of >14 days (χ² = 0.03; p = 0.867). Regarding postoperative complications, the OPB group had a higher rate of severe complications (17.29%) than the NDP group (8.86%), although the difference was not statistically significant (χ² = 2.90; p = 0.089). However, the rates of specific complications, such as hydrothorax, pneumonia, pyothorax, anastomotic fistula, anastomotic stenosis, and 30-day mortality, were similar between the two groups, and no statistically significant differences were observed.

**Table 4:** Surgical evaluation indicators and complications of locally advanced esophageal squamous carcinoma.

Variables	Total (n = 212)	OPB Group (n = 133)	NDP Group (n = 79)	Statistic	*P*
TO, n(%)				χ²=2.88	0.089
Non-TO	171 (80.66)	112 (84.21)	59 (74.68)		
TO	41 (19.34)	21 (15.79)	20 (25.32)		
LND, n(%)				χ²=3.78	0.052
<20	71 (33.49)	51 (38.35)	20 (25.32)		
≥20	141 (66.51)	82 (61.65)	59 (74.68)		
Tumor margin, n(%)				χ²=3.92	**0.048**
R0	199 (93.87)	121 (90.98)	78 (98.73)		
R1	13 (6.13)	12 (9.02)	1 (1.27)		
PLOS, n(%)				χ²=0.03	0.867
>14d	125 (58.96)	79 (59.40)	46 (58.23)		
≤14d	87 (41.04)	54 (40.60)	33 (41.77)		
Complications, n(%)				χ²=3.23	0.072
No	151 (71.23)	89 (66.92)	62 (78.48)		
Yes	61 (28.77)	44 (33.08)	17 (21.52)		
Hydrothorax, n(%)				χ²=11.68	**<.001**
No	194 (91.51)	115 (86.47)	79 (100.00)		
Yes	18 (8.49)	18 (13.53)	0 (0.00)		
Pneumonia, n(%)				χ²=3.15	0.076
No	169 (79.72)	101 (75.94)	68 (86.08)		
Yes	43 (20.28)	32 (24.06)	11 (13.92)		
Pyothorax, n(%)				χ²=1.61	0.205
No	198 (93.40)	122 (91.73)	76 (96.20)		
Yes	14 (6.60)	11 (8.27)	3 (3.80)		
Anastomotic fistula, n(%)				χ²=0.12	0.728
No	195 (91.98)	123 (92.48)	72 (91.14)		
Yes	17 (8.02)	10 (7.52)	7 (8.86)		
Anastomotic stenosis, n(%)				χ²=0.40	0.529
No	206 (97.17)	128 (96.24)	78 (98.73)		
Yes	6 (2.83)	5 (3.76)	1 (1.27)		
Severe complication, n(%)				χ²=2.90	0.089
No	182 (85.85)	110 (82.71)	72 (91.14)		
Yes	30 (14.15)	23 (17.29)	7 (8.86)		
Respiratory failure, n(%)				χ²=2.90	0.089
No	182 (85.85)	110 (82.71)	72 (91.14)		
Yes	30 (14.15)	23 (17.29)	7 (8.86)		
Severe infection, n(%)				χ²=5.82	**0.016**
No	198 (93.40)	120 (90.23)	78 (98.73)		
Yes	14 (6.60)	13 (9.77)	1 (1.27)		
30-days mortality, n(%)				χ²=0.12	0.734
No	207 (97.64)	129 (96.99)	78 (98.73)		
Yes	5 (2.36)	4 (3.01)	1 (1.27)		
30-days readmission, n(%)				χ²=0.00	1.000
No	200 (94.34)	125 (93.98)	75 (94.94)		
Yes	12 (5.66)	8 (6.02)	4 (5.06)		

OPB, other platinum-based, including cisplatin, carboplatin, lobaplatin, and oxiliplatin; NPD, Nedaplatin; TO, textbook outcome; LND, number of lymph node dissection; PLOS, postoperative length of hospital stay.The bold values indicates that this section is statistically significant.

We performed logistic regression to compare the differences in short-term prognostic evaluation indicators between the two groups with covariate adjustments. The results showed that none of the odds ratios (ORs) for the NDP group compared with the OPB group were statistically significant in any of the models, including the endpoints of TO, NCCN-TRG, and JSED-TRG (**Table 5**).

**Table 5:** Covariate adjusted logistic regression with OPB and NDP models in patients with locally advanced ESCC.

Variables	Model1		Model2		Model3		Model4	
	OR (95%CI)	*P*	OR (95%CI)	*P*	OR (95%CI)	*P*	OR (95%CI)	*P*
Endpoint: TO								
OPB Group	1.00 (Reference)		1.00 (Reference)		1.00 (Reference)		1.00 (Reference)	
NDP Group	1.81 (0.91 ~ 3.60)	0.092	2.04 (0.96 ~ 4.36)	0.065	1.79 (0.72 ~ 4.46)	0.214	1.77 (0.64 ~ 4.88)	0.272
Endpoint: NCCN-TRG								
OPB Group	1.00 (Reference)		1.00 (Reference)		1.00 (Reference)		1.00 (Reference)	
NDP Group	1.12 (0.64 ~ 1.95)	0.7	1.15 (0.63 ~ 2.10)	0.659	1.04 (0.53 ~ 2.02)	0.916	0.88 (0.38 ~ 2.04)	0.766
Endpoint: JSED-TRG								
OPB Group	1.00 (Reference)		1.00 (Reference)		1.00 (Reference)		1.00 (Reference)	
NDP Group	1.12 (0.64 ~ 1.95)	0.7	1.15 (0.63 ~ 2.10)	0.659	1.04 (0.53 ~ 2.02)	0.916	0.88 (0.38 ~ 2.04)	0.766

OPB: other platinum-based, including cisplatin, carboplatin, lobaplatin, and oxiliplatin; NPD: Nedaplatin; TO: textbook outcome; TRG: tumor regression grade; OR: Odds Ratio, CI: Confidence Interval.

Model1: Crude.

Model2: Model1 + Adjust: sex, age, HBsAg, Hypertension, Diabetes, family cancer history, smoking, drinking.

Model3: Model2 + Adjust: primary site, weight loss, BMI, albumin, cT, cN, cStage.

Model4: Model3 + Adjust: chemotherapy cycle, radiation approach, Interval between neoadjuvant therapy and surgery, pT, pN, pStage, number of lymph node dissection.

### 3.5 Comparison of long-term prognostic indicators

**Figure 1:**
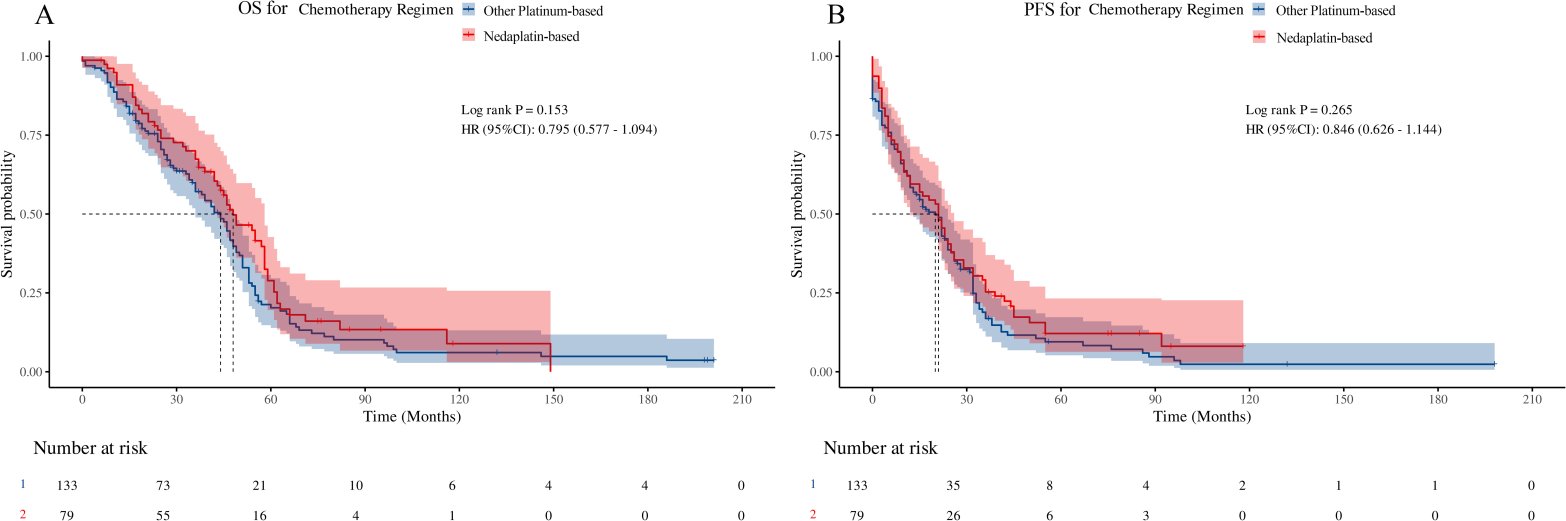
Kaplan-Meier curve and log-rank test based on overall survival (OS) and progression-free survival (PFS) for nedaplatin-based versus other platinumbased regimens. **(A)** OS for chemotherapy regimens; **(B)** PFS for chemotherapy regimens.

**Figure 2:**
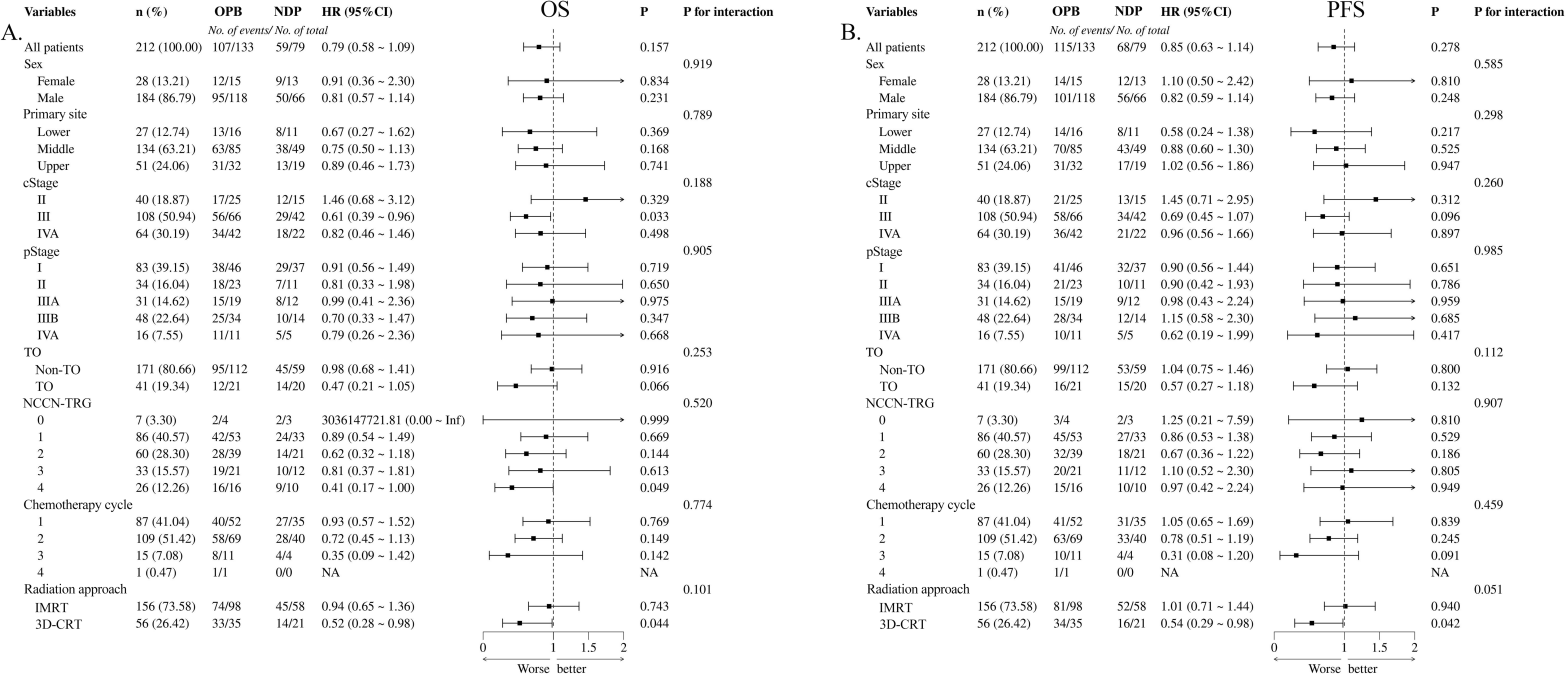
Forest plots based on overall survival (OS) and progression-free survival (PFS) of univariate Cox proportional hazard regression for nedaplatin-based versus other platinum-based regimens. **(A)** OS for chemotherapy regimens; **(B)** PFS for chemotherapy regimens.

PFS and OS were used to assess long-term prognostic efficacy. First, the results of the Kaplan–Meier survival curves and the log-rank test showed that there were no statistical differences between the two groups for either OS (**Figure 1A**, hazard ratio [HR] = 0.795, 95% confidence interval [CI]: 0.577–1.094, p = 0.153) or PFS (**Figure 1B**, HR = 0.846, 95% CI: 0.626–1.144, p = 0.265). Subsequently, we performed univariate Cox proportional hazards regression analyses of each covariate for both groups and plotted the results as forest plots. The forest plots indicated that the OPB group had a worse OS in clinical stage III (**Figure 2A**, 56 deaths of 66 patients in the OPB group, 29 deaths of 42 patients in the NDP group, HR = 0.61, 95% CI: 0.39–0.96, p = 0.033), NCCN-TRG four (**Figure 2A**, 16 deaths of 16 patients in the OPB group, nine deaths of 10 patients in the NDP group, HR = 0.41, 95% CI: 0.17–1.00, p = 0.049), and patients undergoing 3D-CRT (**Figure 2A**, 33 deaths of 35 patients in the OPB group, 14 deaths of 21 patients in the NDP group, HR = 0.52, 95% CI: 0.28–0.98, p = 0.044), and a similarly significantly worse PFS in the population receiving 3D-CRT (**Figure 2B**, 34 of 35 patients in the OPB group, 16 of 21 patients in the NDP group, HR = 0.54, 95% CI: 0.29–0.98, p = 0.042). The p-values for the interactions for all subpopulations did not show statistical differences.

## 4 Discussion

In the present study, we conducted a comprehensive safety and efficacy analysis of neoadjuvant radiotherapy combined with concurrent paclitaxel plus NDP compared with OPB chemotherapy regimens for thoracic segmental ESCC. The safety profile of nedaplatin may be superior to those of OPB agents in terms of acute radiotherapy toxicity and postoperative side effects. However, there was no difference in the efficacy between the two groups in terms of short-term prognostic TRG or long-term OS and PFS. These key findings are important indications for neoadjuvant therapy with NDP for locally advanced ESCC.

The primary focus of this study was the toxic effects and postoperative clinical outcomes of the two treatment regimens. This study found that the toxic effects of neoadjuvant radiotherapy combined with paclitaxel and NDP were generally less severe than those associated with OPB chemotherapy regimens. Specifically, the incidences of ARP and ARE, gastrointestinal reactions, and marrow suppression were significantly lower in the NDP group than in the OPB group. This finding aligns with previous research that highlighted the relatively milder toxicity profile of NDP compared to OPB drugs (23–26). Wang et al. reported 24.5% treatment-related adverse events of grade 3 or worse and Tang et al. observed a significant difference in the incidence of grade 3 and 4 auditory toxic effects favoring nedaplatin over cisplatin (17.7% vs 10.5%, p = 0.04) in patients with nasopharyngeal carcinoma receiving concurrent chemoradiotherapy (16, 27). Several factors may have contributed to the reduced toxicity of NDP. NDP has a unique chemical structure that differentiates it from OPB drugs (28, 29). It contains a monofunctional linker that is believed to affect cellular uptake and intracellular pharmacodynamics, leading to reduced DNA binding and subsequent toxicity. This monofunctional linker may play a role in mitigating the toxic effects of NDP by reducing interactions between the drug and normal tissues (28). Second, the reduced toxicity of NDP may be attributed to its pharmacokinetic properties (29). Studies have shown that NDP has higher lipophilicity and slower renal excretion than OPB drugs, leading to a more sustained plasma concentration and potentially prolonged antitumor activity (27). Kawai et al. have previously indicated that the nephrotoxicity of nedaplatin is associated with its accumulation in the renal cortex, suggesting that while increased lipophilicity may enhance tumor tissue distribution, it could also lead to prolonged renal exposure and increased toxicity risk (30). Jin et al. reported 8.7% of grade 3 anemia leucopenia, 17.4% of grade 4 anemia leucopenia, and 19.6% of neutropenia, respectively, following NDP-based second-line chemotherapy for cisplatin-pretreated refractory metastatic/recurrent ESCC (31). This sustained exposure may allow for more targeted delivery of the drug to tumor cells, thereby reducing the exposure of normal tissues and associated toxicities (32, 33). Furthermore, previous studies suggested that the intracellular processing of NDP may differ from that of OPB drugs, leading to unique mechanisms of action and toxicity profiles (34). For instance, NDP is more susceptible to inactivation by thiol-containing compounds, which may modulate its toxicity and contribute to its milder side effect profile (32, 35). These factors collectively contribute to the favorable toxicity profile of NDP, making it a potentially attractive option for treating thoracic segmental ESCC.

Regarding the postoperative clinical outcomes, the study indicated that while the TO rate was higher in the OPB group than in the NDP group, the difference was not statistically significant. However, the NDP group showed a higher proportion of LNDs with < 20 lymph nodes, suggesting that NDP may have facilitated a more thorough lymph node evaluation. Moreover, R0 tumor margin rates were significantly higher in the NDP group, indicating a greater likelihood of achieving clear surgical margins and potentially better oncological outcomes. Ohnuma et al. also reported NDP-based nCRT had an R0 resection rate of 89.3% and a pathological complete response rate of 32% in patients with locally advanced ESCC (36). Higher lymph node dissection rates may suggest improved surgical thoroughness, potentially contributing to higher R0 resection rates and better oncological outcomes. Supporting this, Shang et al. have previously demonstrated that extensive lymph node dissection correlates positively with improved survival and reduced recurrence rates (37). The PLOS was similar between the two groups, with a higher proportion of patients in both groups having a PLOS of > 14 days. This suggests that NDP does not adversely affect the duration of the hospital stay after surgery. The OPB group exhibited a higher rate of severe postoperative complications than the NDP group, although this difference was not statistically significant. Specifically, the rates of hydrothorax, pneumonia, pyothorax, anastomotic fistula, anastomotic stenosis, and 30-day mortality were similar between the two groups, indicating that NDP did not increase the risk of these specific complications. The reduced toxicity associated with NDP may contribute to its potential benefits on postoperative outcomes, as it allows for better patient tolerance and recovery from chemotherapy and radiotherapy (38–41). Thus, the present study suggests that neoadjuvant radiotherapy combined with concurrent paclitaxel and NDP may offer some advantages in terms of postoperative evaluation indicators compared with OPB chemotherapy regimens.

In terms of short- and long-term prognosis, the findings suggest that, in the context of this study, the use of NDP in neoadjuvant chemotherapy does not lead to significant improvements in short-term prognostic evaluation indicators compared to OPB drugs. This may be due to the similar efficacy profiles of NDP and OPB in the treatment of thoracic segmental ESCC as well as the complex interplay of various factors that affect prognosis, such as tumor stage, lymph node status, and patient characteristics (42–45). In the long-term prognostic analysis, Kaplan–Meier survival curves and log-rank tests showed no significant differences in the OS or PFS between the two groups. However, the subgroup analysis revealed that the OPB group had a worse OS in patients with clinical stage III, NCCN-TRG four, and 3D-CRT. Similarly, the OPB group had a significantly worse PFS than the 3D-CRT group. Isohashi et al. previously presented that IMRT had better locoregional control, PFS, and 3-year OS rate than 3D-CRT (95% vs. 85%, 92% vs. 70%, 92% vs. 85%, respectively) in patients with cervical cancer receiving NDP-based treatment (46, 47). The lack of significant differences in the OS and PFS between the two groups may be due to the small sample size and the potential for residual confounding factors that could influence survival outcomes. It is also possible that the benefits of NDP in terms of reduced toxicity and improved patient tolerance, which may contribute to better adherence to the treatment regimen and overall treatment efficacy, are not fully reflected in the OS and PFS outcomes (48–50). Shukuya et al. observed significantly better OS in the NDP group (median 13·6 months, 95% CI 11·6-15·6) than in the cisplatin group (11·4 months,10·2-12·2; hazard ratio 0·81, 95% CI 0·65-1·02; p=0·037) with fewer grade 3 or worse nausea (3.95% vs. 14.29%), fatigue (3.39% vs. 11.43%), hyponatremia (13.56% vs. 30.29%), and hypokalemia (2.26% vs. 8.57%) in advanced squamous cell lung cancer (12). Besides, the significant difference in age between the two groups suggests that it may affect chemotherapy tolerance. Vilmi et al. have previously support using less toxic platinum-based regimens, such as nedaplatin, for elderly patients to minimize adverse events (51). Therefore, while NDP appears to have a favorable toxicity profile and may contribute to better postoperative outcomes, the study did not demonstrate significant differences in short- or long-term prognostic indicators when compared with OPB.

The limitations of this study include its retrospective design, which may introduce selection bias; a small sample size, particularly in the NDP group, which limits generalizability; and the lack of molecular profiling data, precluding a deeper understanding of the molecular mechanisms underlying treatment response and outcomes. Future work should include prospective randomized trials to confirm the efficacy and safety of neoadjuvant radiotherapy with NDP in a larger patient population, incorporate molecular profiling to elucidate the role of specific biomarkers in predicting the response to NDP-based neoadjuvant therapy, and further investigate long-term outcomes, including quality of life and survival, to fully assess the clinical utility of NDP in the treatment of thoracic segmental ESCC.

## 5 Conclusions

In conclusion, this retrospective study compared the safety and efficacy of neoadjuvant radiotherapy combined with paclitaxel plus NDP with those of OPB chemotherapy regimens for thoracic segmental ESCC. The findings revealed that NDP exhibited a favorable toxicity profile with reduced acute radiotherapy toxic effects and postoperative side effects compared with OPB drugs. However, the efficacy outcomes, including pathological response rates, OS, and PFS, did not differ significantly between the NDP and OPB chemotherapy groups. These findings contribute to a better understanding of the potential advantages and limitations of NDP in the context of neoadjuvant therapy for locally advanced ESCC and guide the development of personalized and effective treatment strategies tailored to the specific needs of patients with thoracic segmental ESCC.

## Data Availability

The data presented in this study are available on request from the corresponding author due to protection of patient privacy.
